# An integrated 4249 marker FISH/RH map of the canine genome

**DOI:** 10.1186/1471-2164-5-65

**Published:** 2004-09-13

**Authors:** Matthew Breen, Christophe Hitte, Travis D Lorentzen, Rachael Thomas, Edouard Cadieu, Leah Sabacan, Allyson Scott, Gwenaelle Evanno, Heidi G Parker, Ewen F Kirkness, Ruth Hudson, Richard Guyon, Gregory G Mahairas, Boris Gelfenbeyn, Claire M Fraser, Catherine André, Francis Galibert, Elaine A Ostrander

**Affiliations:** 1 Department of Molecular Biomedical Sciences, College of Veterinary Medicine, North Carolina State University, 4700 Hillsborough Street, Raleigh, NC 27606, USA; 2 UMR 6061 CNRS, Génétique et Développement, Faculté de Médecine, 35043 Rennes Cédex, France; 3 Clinical and Human Biology Divisions, Fred Hutchinson Cancer Research Center, Seattle WA 98109-1024, USA; 4 The Institute for Genomic Research, 9712 Medical Center Drive, Rockville, MD, 20850, USA; 5 Oncology Research, Animal Health Trust, Lanwades Park, Suffolk, CB8 7UU, U.K; 6 VieVax Corp. 1616 Eastlake Ave. E., Seattle, WA 98102 USA

**Keywords:** canine, dog, radiation hybrid, microsatellites, ESTs, BAC-ends

## Abstract

**Background:**

The 156 breeds of dog recognized by the American Kennel Club offer a unique opportunity to map genes important in genetic variation. Each breed features a defining constellation of morphological and behavioral traits, often generated by deliberate crossing of closely related individuals, leading to a high rate of genetic disease in many breeds. Understanding the genetic basis of both phenotypic variation and disease susceptibility in the dog provides new ways in which to dissect the genetics of human health and biology.

**Results:**

To facilitate both genetic mapping and cloning efforts, we have constructed an integrated canine genome map that is both dense and accurate. The resulting resource encompasses 4249 markers, and was constructed using the RHDF5000-2 whole genome radiation hybrid panel. The radiation hybrid (RH) map features a density of one marker every 900 Kb and contains 1760 bacterial artificial chromosome clones (BACs) localized to 1423 unique positions, 851 of which have also been mapped by fluorescence *in situ *hybridization (FISH). The two data sets show excellent concordance. Excluding the Y chromosome, the map features an RH/FISH mapped BAC every 3.5 Mb and an RH mapped BAC-end, on average, every 2 Mb. For 2233 markers, the orthologous human genes have been established, allowing the identification of 79 conserved segments (CS) between the dog and human genomes, dramatically extending the length of most previously described CS.

**Conclusions:**

These results provide a necessary resource for the canine genome mapping community to undertake positional cloning experiments and provide new insights into the comparative canine-human genome maps.

## Background

Three major advances in the development of resources for mapping canine disease genes have been: 1) the development of a radiation hybrid (RH) map composed of large numbers of microsatellite markers and genes that link the canine and human genomes [1], 2) the development of canine specific whole chromosome paints that have allowed preliminary assignment of conserved segments between human and dog [3-5]; and 3) the publication of a 1.5x genome sequence of the dog [2]. The most recently published RH map of the dog comprises 3270 markers including 1596 microsatellite-based markers, 900 canine-specific cloned gene sequences and expressed sequence tags (ESTs), and an initial set of 668 canine-specific BAC-ends [1]. The map was constructed using the RHDF5000-2 whole genome radiation hybrid panel [6] and features markers mapped to 3009 unique positions, defining an average inter-marker distance of one megabase (Mb). The map also defines a minimal screening set of 325 highly informative well-spaced markers, to be used in the initiation of genome-wide scans [1]. A well-defined synteny between the dog and human genomes was established as a function of this work [1] and from extensive reciprocal chromosome painting studies [3,5,7].

The above mapping efforts are complemented by the recent release of a 1.5x sequence of a Standard Poodle genome [2]. The sequence includes 6.2 million sequence reads that span approximately 78% of the genome. More than 650 million base pairs (>25%) align uniquely to the human genome, and the resulting alignment includes fragments of putative orthologs for 18,473 of 24,567 annotated human genes. The current alignment supports most of the proposed comparative segments, but suggests that the final comparative dog-human map will be composed of at least 160 comparative blocks [2].

Using the above resources, canine researchers have undertaken genome-wide screens for linkage to a variety of disease loci as well as morphological traits defining differences between breeds. Many of these studies have met with success. Disease loci have been genetically mapped or otherwise localized in the dog for several disorders including: vision-associated disease such as progressive rod cone degeneration, early retinal degeneration, cone degeneration, and collie eye anomaly [8-11], kidney cancer [12,13], narcolepsy [14], rheumatoid arthritis [15], Severe Combined Immunodeficiency (SCID) [16], hip dysplasia [17], cystinuria [18], bleeding disorders [19,20], ceroid lipofuscinosis [21], and copper toxicosis [22,23]. In addition, quantitative trait loci (QTL) have been identified for principal components defining skeletal variation and hip dysplasia [17,24]. In general, investigators have been able to use the existing resources to localize traits of interest to an interval of 10–20 Mb, but in most cases the causative gene remains to be identified.

To facilitate positional cloning efforts in the canine community, we have localized a large set of randomly selected canine-specific BACs onto the canine 5000-rad RH map, a subset of which we have also localized cytogenetically. The resulting map of 4249 markers includes 1760 mapped BAC-ends, 851 of which have been FISH mapped, to generate a dense, accurate and highly integrated map of the canine genome.

## Results

### General RH map characteristics

This novel RH map of the dog genome contains 4249 markers of three different types: 900 genes, 1589 microsatellites and 1760 BACs. The map was generated by genotyping 1092 new BAC-end markers on the RHDF5000-2 panel, and recomputing the new vectors with those from the previous map [1] using MULTIMAP [25] and TSP/CONCORDE [26]. Table 1 summarizes the key features of the new map. The typing of 4249 markers resulted in an RH map containing 4106 markers that were eventually grouped and assigned to each of the canine chromosomes, leaving only 143 unlinked markers. Human orthologs were identified for 2233 mapped markers. The resolution limit of the RHDF5000 panel has been determined to be 4 cR5000 (600 Kb) [6]. Thus, markers falling within any 4 cR5000 or 600 Kb stretch cannot be ordered relative to one another with high confidence and, consequently, are reported as co-localized on the map.

**Table 1:** Key features of the integrated canine RH/FISH map

		**Number of**							
		
		**Markers**	**Unique**	**BACs**	**Unique**	**BACs**	**BACs**		
**Chromosome**	**Size (Mb)^(1)^**	**RH Mapped**	**RH Positions^(2)^**	**RH Mapped**	**BAC RH Positions**	**FISH Mapped**	**RH/FISHed in Common**	**Human Coordinates^(3)^**	**Human/ Dog CS^(4)^**
CFA01	137	220	169	104	83	60	48	110	4
CFA02	99	140	99	55	43	37	27	78	**4**
CFA03	105	155	123	74	60	46	35	71	3
CFA04	100	147	106	60	51	37	29	80	3
CFA05	99	158	102	76	53	40	28	74	4
CFA06	87	122	69	53	35	29	19	69	3
CFA07	94	173	107	78	56	44	37	97	2
CFA08	86	135	98	64	50	30	25	77	1
CFA09	77	124	93	39	33	31	26	78	2
CFA10	80	121	91	53	41	28	22	68	3
CFA11	86	134	116	56	49	38	36	78	2
CFA12	85	181	127	71	55	32	29	94	1
CFA13	75	93	65	38	32	21	18	53	2
CFA14	72	114	85	54	42	31	28	62	2
CFA15	75	113	94	42	38	26	22	63	5
CFA16	73	93	79	42	36	23	23	44	3
CFA17	80	130	96	53	41	20	18	69	2
CFA18	66	118	99	50	45	30	27	71	2
CFA19	66	96	66	40	31	18	18	49	2
CFA20	66	139	95	52	41	20	16	74	2
CFA21	61	103	86	34	31	26	22	60	1
CFA22	61	113	76	52	33	17	15	70	1
CFA23	61	79	67	40	36	23	21	45	1
CFA24	73	84	66	41	33	18	16	42	1
CFA25	60	94	75	41	36	27	22	56	4
CFA26	48	85	66	39	33	19	13	44	3
CFA27	57	94	76	29	27	19	16	50	1
CFA28	55	84	70	39	35	26	24	47	1
CFA29	51	81	71	33	31	20	18	42	1
CFA30	47	63	50	26	21	18	16	38	1
CFA31	50	53	47	25	23	16	13	25	2
CFA32	51	56	46	33	28	14	15	27	1
CFA33	41	63	52	30	28	20	20	40	1
CFA34	50	65	49	23	13	15	15	47	2
CFA35	38	49	35	24	16	14	12	30	1
CFA36	41	61	52	26	26	13	13	40	1
CFA37	40	65	45	27	21	20	17	37	1
CFA38	38	32	28	16	14	9	9	16	1
CFAX	139	66	44	26	21	23	22	28	1
CFAY	27	10	10	2	2	2	1	1	1
unlinked		143							
TOTAL	2797	4249	3090	1760	1423	1000	851	2233	79
Average Distance (Mb) Between Markers	--	0.66	0.91	1.59	1.97	2.80	3.48	1.25	

**Legend: Map statistics.**^(1)^ Chromosome size in Mb based on bivariate flow cytometry measurements

^(2)^ Unique RH positions with one or more markers

^(3)^ Human coordinates identified through BLAST analysis (see Methods)

^(4)^ Conserved segment identified by RH mapping with 2 or more loci

Analysis of the entire dataset of 4249 RH vectors by MULTIMAP [25] at a Lod score of 8.0, and for a subset of regions a Lod of 9.0, resulted in 60 individual linkage groups that could be assigned to the 38 canine autosomes and two sex chromosomes. For chromosomes that were covered by multiple linkage groups, the data were merged and ordered as described in the Methods. FISH data combined with significant Lod scores between selected markers led to the unambiguous assignment and orientation of specific linkage groups to chromosomes and, ultimately, complete coverage of each chromosome.

**Figure 1:**
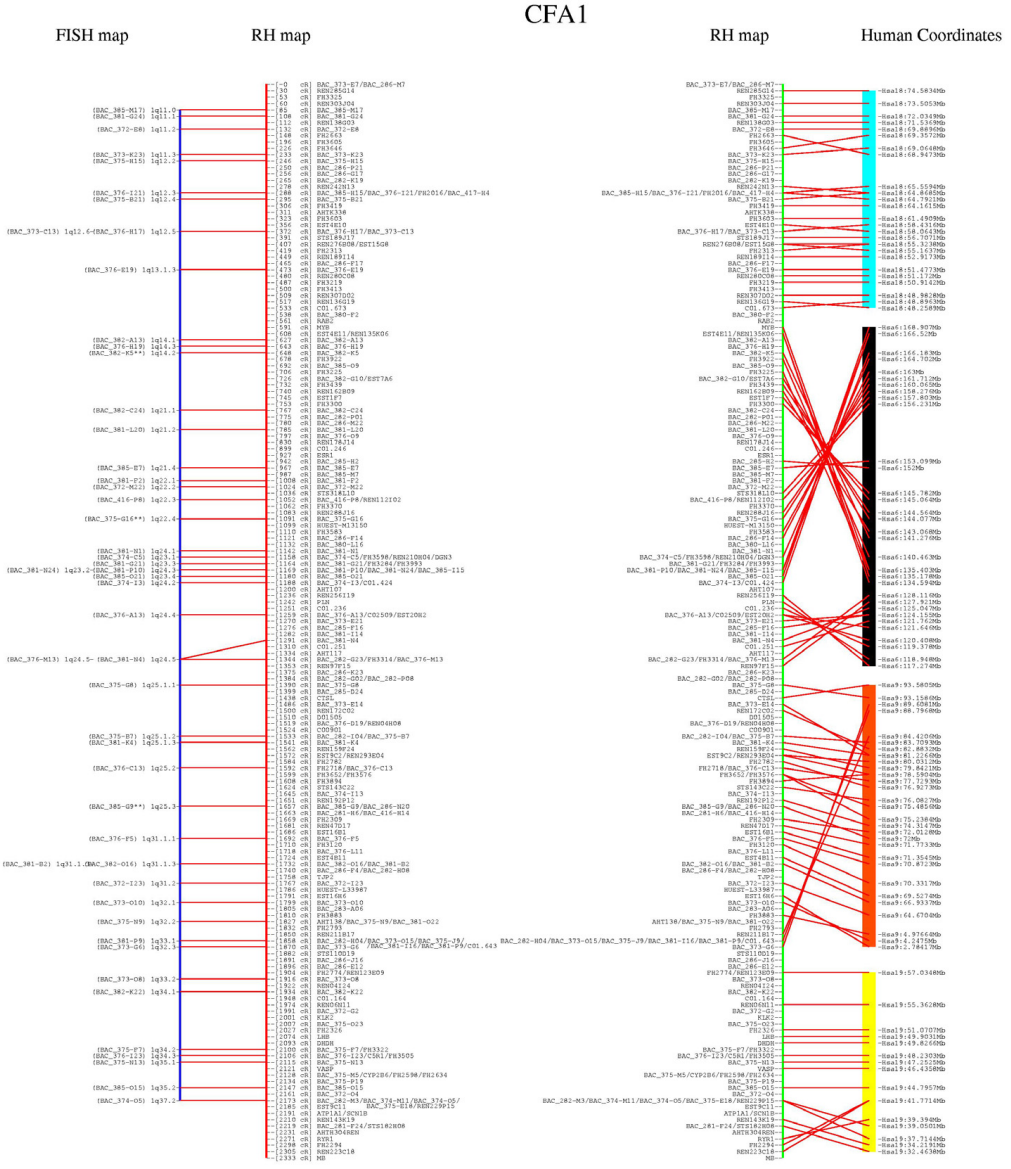
**Integrated FISH/RH map and dog/human comparative data for CFA1. **BAC-ends also localized by FISH mapping are reported in the left panel, symbolized by a vertical bar along with BAC addresses. Asterisks (**) represent BAC clones that did not have a unique cytogenetic location (multiples hits are listed on SOM). RH mapped markers and their cumulative positions in centiRay 5000 units are reported to the right of the FISH map. Connecting lines between FISH and RH maps indicate integration points between maps. The right panel shows the human evolutionarily conserved segments, represented by colored boxes as determined by RH data. Human coordinates (in Mb) identified from dog/human sequence alignments (see Methods) are reported on the right most vertical bar. RH markers and their corresponding human sites are connected by lines and illustrate the rearrangement within synteny blocks.

The resulting 4106 linked markers defined 3090 unique positions regularly spaced across all autosomes. The largest chromosome, canine chromosome 1 (CFA 1), contains 220 markers, while CFA 38, the smallest chromosome at 38 Mb, has only 32 markers (Figure 1). Excluding the sex chromosomes, the least dense chromosome, CFA 38, has a marker located every 1.2 Mb, while the most dense, CFA 12 and CFA 20, have markers positioned at an average of every 0.47 Mb. The increase in density is due solely to the addition of 1092 new BAC-ends, bringing the total to 1760 from the 668 reported in the previous version of the map. The mean interval size is now 1.59 Mb. The chromosome with the most BACs mapped to it is CFA 1, featuring 104 localized to 83 unique positions (Figure 1). See all chromosomes in the Supporting Online Material (SOM) at . Again, the chromosome with the fewest is CFA 38, with 16 BACs localized to 14 unique positions. This resource provides a dense scaffold on which to build regional physical maps and search for new genes. Of particular interest to disease gene mappers will be the 26 BACs that are RH mapped to the X chromosome, 13 of which are also localized by FISH. Only two BACs were localized to the Y chromosome, one of which is also FISH mapped. See Table 1 for details.

### FISH mapped BACs

**Figure 2:**
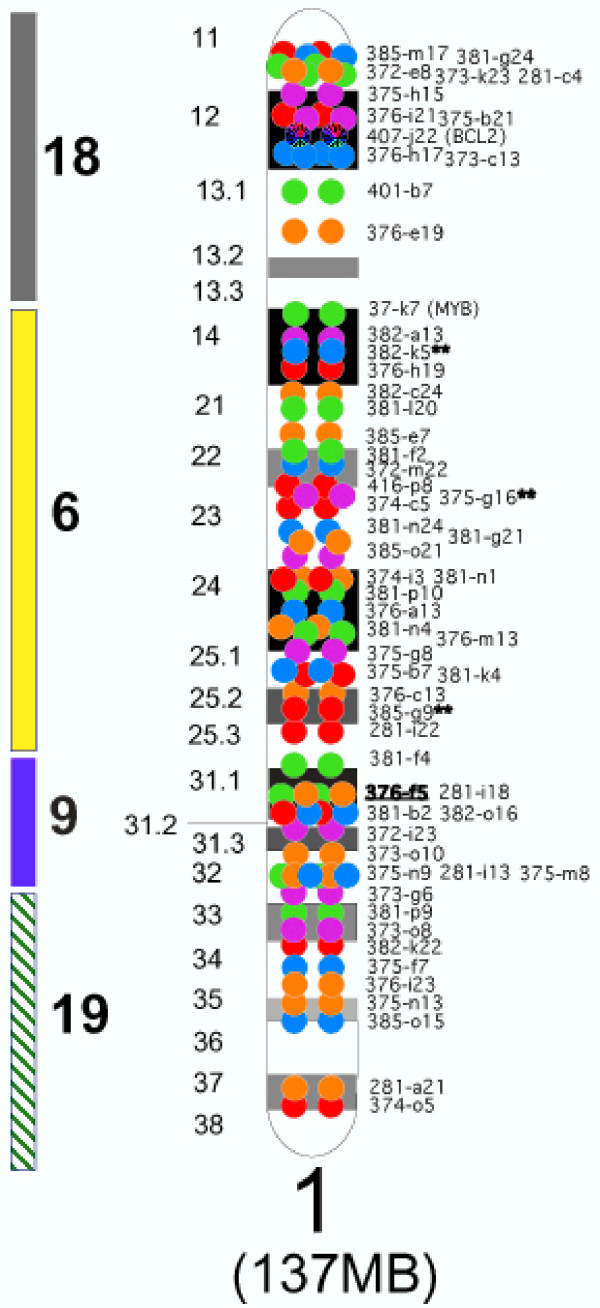
**Assignment of 60 canine BAC clones to CFA 1. **BAC addresses alongside the ideogram of CFA 1 refer to clones from the RPCI-81 canine BAC library. A pair of colored spots spanning an interval of approximately 4 Mb represents the cytogenetic assignment of each clone. The color of the spots identifies the fluorochrome used to label the clone as follows: red = Spectrum Red; orange = Spectrum Orange; green = Spectrum Green; blue = DEAC; purple = Cy5. Multi-color FISH of neighboring clones, using both metaphase and interphase analyses, was used to establish the precise order of the clones along the length of the chromosome. Clones whose assignment is represented by a circular rainbow have been tentatively placed, but not yet co-localized with neighboring clones to establish one equivocal, linear order. BAC addresses followed by ** identify those clones that resulted in fluorescent signal at more than one location. Human orthologous regions (HSA) are reported on the left of the figure by vertical bars.

A total of 1,000 BACs were assigned to a chromosome band, and then ordered along the length of the corresponding chromosome using a combination of metaphase and interphase multi-color FISH. CFA 1 is shown, for example, in Figure 2. The panel of FISH mapped clones included clones representing both ends of each chromosome, with the exception of the centromeric end of CFA 6 (6q11-q12) and the centromeric 20% of CFA 9 (9q11-q12). Nine hundred and eighty-one out of 1,000 BACs had a unique cytogenetic location, and 851 were also ordered on the RH map. This yielded an average of one BAC-end that was both FISH and RH mapped every 3.48 Mb. See SOM material for data on all chromosomes and .

### Integration of FISH and RH data

Analysis of co-linearity between the two maps was performed by connecting the BAC markers in common between the RH map and FISH data. This approach allowed the identification of markers that serve as anchors to each chromosome for both RH and FISH maps, thus validating their localization and marker order. Early in the assembly process, a small number of discrepancies between the two maps were easily identified through graphical drawings of all chromosomes, leading to a systematic re-examination of experimental data and, in most cases, consensus resolution. However, 19 BACs did not have one unique cytogenetic location. These are indicated with a double asterisk in Figure 1 and 2, as well as the SOM and figures provided on all web sites. For each of these 19, one of their FISH localizations was in agreement with the corresponding RH map position. In addition, seven (7/19) had an identified human ortholog that was in agreement with the RH map localization. For an additional set of 24 BACs, conflict remained after rechecking both the FISH and RH data. These are indicated by "#" in all Figures. Interestingly, the DNA used to obtain FISH data for these clones PCR amplified successfully with the same primers used to obtain RH data, indicating that the DNA samples were the same and had not undergone sample mix-up. Finally, a human ortholog sequence was identified that was in agreement with the RH data for 15 (15/24) of these clones.

Although we have no clear explanation for these discrepancies, it is of note that the two mapping methods used different DNA sequences to define the same marker. In the RH method, a pair of 25-mer oligonucleotides defining only one end of the dog insert DNA is used. By comparison, in the FISH analysis, the whole BAC encompassing approximately 150 Kb is hybridized. Since the DNA for the FISH localization was prepared from cultures initiated from single bacterial colonies, these anomalies suggest that at least a subset of the clones may be chimeric. An additional explanation is that the clones are hybridizing to genomic regions containing one member of a multi-gene family. Whatever the explanation for these discrepancies, it is noteworthy that the total number of discrepancies between the FISH and RH data represent 3% of the total data. This is in agreement with reports that the frequency of chimeric clones in this BAC library is very low [27]. Thus, in most cases, the co-linearity of the FISH and RH maps is perfect, i.e. the order of the BACs determined by multi-color FISH is identical to that derived from analysis of genotyping data using primers defining single BAC-ends.

In addition to the conflicting results described above, in some cases, we note that the order for two closely positioned markers is inverted between the FISH and the RH data. However, for all of these cases the distance between the two markers is estimated to be less than 50–100 Kb, as these clones overlap in interphase nuclei. Thus, they are well within the 600 Kb resolution limit of the RH5000 panel. Such minor inaccuracies in marker order will not be detrimental to gene mapping, as the integrated map has fixed positions every 3.42 Mb with support from FISH data. The example of CFA 1 is provided (Figure 1 and 2). Please refer to SOM and websites for all other chromosomes.

### Synteny and conserved segments

Two criteria were used to assign dog BAC clones to orthologous locations of the human genome. First, when compared to the complete human genome, each component of the paired end-sequences had greatest similarity to locations that are separated by 50–500 Kb. Although most BAC clones have inserts of 100–200 Kb, minor differences between the two genomes such as local duplication or loss of specific genes should be accommodated by the 50–500 Kb range. If one considers a single aligned end-sequence, the probability of the paired end-sequence aligning spuriously within 500 Kb in the human genome is approximately 0.03%. The second criterion was that each pair of end-sequences should align in a head-to-head orientation. With this additional condition, the probability of scoring pairs that contain spurious alignments falls to less than 0.01%.

In the most recent version of the map [1], 75 conserved segments (CS) were detected within the 38 canine autosomes. In addition, two CS corresponding to the sex chromosomes and nine singletons were detected. These 77 CS were identified through the analysis of 820 canine markers having an unambiguous ortholog in the human sequence. In the present map, that number is more than doubled with 2233 markers having an ortholog in the human sequence. These newer data confirm all previously described CS, and incorporate two singletons into two novel CS for a new total of 79 CS. For the sake of clarity, the remaining singletons were not reported in this new version of the map.

Interestingly, while the number of CS did not change significantly compared to the previous iteration of the RH map, their nature and composition were altered, as shown for CFA 1 (Figure 1). Four CS were identified for CFA1 that correspond, in order from centromere to telomere, to human chromosomes (HSA) 18, 6, 9 and 19. From the density of markers for which an ortholog can be identified, it is clear that the order of markers is consistent between CFA 1 and HSA 18. Thus, this CS would be considered a "conserved and ordered segment" (CSO). For the CS corresponding to HSA 6, however, two sub-segments are noted which are caused by an inversion. In each of these two sub-segments, the order of markers is comparable to the syntenic portion of HSA 6, and each canine segment would independently be considered a CSO.

In addition to identification of some previously unknown CSO, the increased number of markers for which a human ortholog could be identified unambiguously allowed for a substantial increase in the size of many previously identified CS. At present, as shown in Figure 1 and SOM, CS in the dog are highly contiguous with the human genome. Indeed, only a few markers identified as human orthologs fall outside of a CS. This significantly refines our knowledge of evolutionary breakpoints between the dog and human genomes.

## Discussion

The 4106 markers that constitute this new map occupy 3090 distinct positions, with an average of 1.3 markers per position. This reflects the fact that with 4106 genotyped markers, we are approaching the saturation level of 4500 markers predicted on the basis of size of the canine genome, and resolving power of the 5000 rad panel [6]. A detailed analysis of the co-localized markers indicates that in the majority of cases markers are co-positioned with other markers of different types, i.e. BACs and microsatellites, or microsatellite and genes, etc. Thus, all of the data will likely prove useful for mapping and cloning genes of interest, as the information provided at each location is non redundant. In addition, even when markers of the same type are co-localized, they are likely to be of value; any given microsatellite is not informative in every pedigree, and closely localized BACs may represent the beginning of an overlapping contig.

A direct consequence of markers mapping to the same positions is that the mean distance between two adjacent positions is now 0.9 Mb, which is very similar to the 1 Mb calculated for the most recent published version of the canine map [1]. Again, this reflects the resolving power of the 5000 rad panel [6], as well as the method of map computation. TSP/CONCORDE considers markers co-localized if they map to a region of 0.9 Mb or less, regardless of RH panel mapping power [26].

The number and identity of conserved segments (CS) between the human and the dog genomes is 79. CS were identified by both FISH localization and RH mapping with four chromosomes showing four human/dog CS, and the remainder showing one to three. The number of markers and the resolving power of the 5000 rad panel allowed us to detect several instances in which CS appear to be split in sub-segments. In several such cases, the order of markers in the two adjacent sub-fragments is conserved, although the two sub-fragments are oriented in opposing directions. Following the previously agreed definitions, these sub-fragments are termed CSO for "conserved segment order" [28]. Such a situation is observed in CFA 1 (Figure 1). In other instances, one of the two adjacent sub-fragments appears to correspond to a CSO, while the other corresponds to a CS. Obviously a better distinction between CS and CSO segments would require genotyping of additional markers, ideally on a panel constructed with a higher dose of radiation, and with concomitantly higher resolution. Such experiments are currently underway using 10,000 genes derived from the available dog genome sequence [2] and a newly constructed 9000 rad panel (data not shown).

As shown in Table 1, and as indicated in the Results, 851 BACs were localized both by FISH and RH mapping, with a high level of concordance between the two methods. Thus, the map presented here provides a verified anchor point every 3.48 Mb. Unlike a FISH map that is not altered or modified by the addition of a new dataset, an RH map does change after adding information because different algorithms provide different solutions for local marker order. Each is statistically valid when ordering the same set of markers. However, discrepancies may arise as more markers are added to the map. Moreover, even one algorithm can generate maps with slightly different marker orders within localized regions, even when only minor adjustments are made to the dataset like removing seemingly redundant markers [29]. In the present study, the high degree of anchorage by a set of markers localized in different laboratories with differing technologies yields a high level of confidence for the integrated FISH/RH map.

Of interest to many researchers will be the multiple applications that a cytogenetically defined and RH verified set of canine BAC clones can provide. For example, the ordered set of 981 FISH mapped canine BAC clones with a unique cytogenetic location will be welcomed by cancer geneticists as a means to characterize chromosome aberrations in canine tumors. The dense cytogenetic coverage available for most chromosomes allows the use of serial differential labeling of some or all clones in chromosome-specific panels designed to 'tile' structurally aberrant chromosomes. This approach will allow a much more accurate assessment of chromosome rearrangements than is possible with single color whole chromosome paint probes. In addition, the ordered clones will allow expansion of the current canine BAC microarrays, which in turn may be used for a variety of applications including array-based Comparative Genome Hybridization (CGH) analysis of canine tumors [30], array-painting of aberrant canine chromosomes, and investigation of cytogenetically cryptic copy number changes and gene dosage alterations in congenital abnormality syndromes. In addition, ordered arrays of BAC clones will be an important resource for refining the comparative cytogenetic data within the diverse karyotypes that comprise the *Canidae*. Finally, with the imminent release of the 7x canine genome assembly, the resources described in this paper will provide a long-awaited means to translate canine cytogenetics data into canine DNA sequence data, thus advancing our knowledge of canine and comparative genomics.

## Conclusions

The presentation of a map containing 900 canine specific genes, 1589 microsatellites and 1760 BACs provides the canine genetics community with nearly all the resources it needs to undertake experiments aimed at both mapping and cloning traits of interest. A dense set of microsatellite markers (MSS-2) for undertaking genome wide scans was provided in the previous iteration of the map [1]. These same markers are integrated into the current map. Thus, within 0.7/0.8 Mb of any linked marker on the current map is now an array of anchored BACs for contig building, comparative mapping, and searching for new genes and splice variants. The current map defines some 79 conserved segments between human and dog. While we expect this number to approximately double when the 7x canine genome sequence is completed, we speculate that given the density of markers mapped and the distance between adjacent CS, new CS defined by the 7x sequencing effort will be short in size and harbor a limited number of genes. What remains in canine genome map building is the development of a very high resolution gene map that can assist in the assembly of the 7x canine genome sequence now underway, and provide a mechanism for moving easily between canine and human comparative segments. This is currently being undertaken. In the meantime, canine researchers can move forward with the continued development of the dog model for mapping and cloning genes of interest to both human and companion animal health.

## Methods

### Markers and primer selection

Canine BACs were randomly selected from the RPCI-81 canine BAC library [27] and were end-sequenced as described previously [1]. DNA was prepared using standard automated approaches [31] and sequenced either at The Institute for Genomic Research (TIGR) or the University of Washington High Throughput Sequencing Center. Average read lengths were in excess of 700 bp. Trace files representing BAC-end sequences were imported from ABI sequencers and examined for sequence homology to cloning vectors, *Escherichia coli *(*E. coli*) and repetitive DNA sequences. BAC sequences were also compared using Cross_Match with the complete *E. coli *genome to remove contaminating sequences of bacterial host origin. Finally, sequences were examined for interspersed repeats and regions of low sequence complexity using RepeatMasker . Primers defining each BAC-end were designed to regions of high quality sequence data using Primer3 software . Primers of 25 bp lengths were preferentially designed in order to minimize problems associated with non-specific amplification, to generate amplicons of 200 to 500 bp, and to work under a single optimal set of PCR conditions.

### Genotyping

Genotyping was performed using the RHDF5000-2 panel, which is comprised of 118 cell lines. The panel was constructed by fusing dog fibroblasts irradiated at 5000 rads with TK-HTK3 hamster cells and has an experimental retention frequency of 22% with a theoretical resolution limit of 600 Kb [32].

PCR reactions were carried out at the University of Rennes and the Fred Hutchinson Cancer Research Center (FHCRC) as described previously in 15 μl volumes [1,33-35] using the following touchdown program: 8 min 95°C, followed by 20 cycles of 30 sec 94°C, 30 sec 63°C decreasing by 0.5°C per cycle, 1 min 72°C and 15 cycles of 30 sec 94°C, 30 sec 53°C, 1 min 72°C and a final extension of 2 min 72°C. Primer pairs yielding either faint or spurious bands were rejected. PCR products were resolved and recorded as described [33,35] through a semi-automated data acquisition software.

### Quality control

Duplicate genotypes were obtained for the 1092 new BACs added to the map. Data were considered consistent when the number of discrepancies between data sets was ≤ 16%, a value determined to correspond to a distance lower than the resolution limit of the RHDF5000-2 panel. In the rare cases where two independent typings yielded >16% discrepancies, a third typing was done and the resulting vector was either integrated into the map construction, or the marker was discarded if no agreement was observed between two of three genotypes.

### RH map construction

RH vectors corresponding to the BAC-end markers and marker vectors of the previous map [1] were computed as a single data set using the MultiMap and TSP/Concorde algorithms [1,25,26,29]. Linkage groups were generated initially at a Lod score of 8.0, and where needed, at 9.0 to ensure strong statistical support.

Individual linkage groups were analyzed using the multipoint approach of the rh_tsp_map version 2.0 of TSP/CONCORDE, as described previously [1,29]. Inter-marker distances are expressed in cR5000 units. Framework maps of well-spaced markers, supported by high quality data between adjacent markers, were initially generated for all chromosomes. Distance criteria were set to 4cR, corresponding to the resolution capacity of the RH panel used, and quality criteria were fixed to 4, corresponding to a maximum allowable number of ambiguous data within RH vectors, as determined from previous analyses [1,35]. Distance and ordering of markers within each group was then determined by the TSP/Concorde and rh_tsp_map-2 algorithms [26,29]. Markers that could not be ordered with a high confidence level were submitted for re-analysis by stepwise increases in the Lod score to > 9.0, forcing the linkage group to split into two or more groups, until satisfactory order with a high statistical confidence level was achieved for each resulting group. Groups were then merged and oriented into a unique dataset. The merging step utilized the cytogenetic data obtained as part of this study, as well as the 2-point Lod scores between the markers at the extremes of each linkage group. Lod scores were generated using the pairlod_dist software from the rh_tsp_map package [26].

### FISH mapping

A total of 851 BAC clones from the RH map were also localized by multi-color FISH analysis. DNA from each clone was prepared from 2.5 ml cultures using a BAC RealPrep (Qiagen, Valencia, CA) protocol. Two hundred nanograms from each sample were labeled using nick translation to incorporate one of five fluorochromes, Spectrum Red/Orange/Green dUTP (Vysis, Downers Grove, IL), diethylaminomethylcoumarin (DEAC)-5-dUTP (NEN/Perkin Elmer Life Sciences, Boston, MA), or Cy5-dUTP (Amersham Biosciences, Piscataway, NJ). Typically, 25 ng of each of five differentially labeled probes were pooled and precipitated in the presence of 15 μg of sonicated genomic dog DNA as competitor. Chromosome preparation, probe hybridization and post hybridization washes were performed as described previously [35,36]. Chromosomes were counterstained in 80 ng/ml 4', 6-diamidino-2-phenylindole (DAPI) and mounted in anti-fade solution (Vectashield, Vector Laboratories, Burlingame, CA). Images were acquired and processed using a multi-color FISH workstation comprising a fluorescence microscope (Axioplan 2ie, Zeiss) equipped with narrow pass filter sets and a cooled CCD camera (CoolSnapHQ, Photometrics, Tuscon, AZ) both driven by dedicated software (SmartCapture 2.3.1 Digital Scientific, Cambridge, U.K.). The digital image of each DAPI stained metaphase spread was processed using a high-pass spatial filter to reveal enhanced DAPI bands. Clones were assigned to a chromosome region according to the DAPI banded nomenclature of Breen et al. [35,36]. Refinement of probe order along the length of each chromosome was made by subsequent rehybridization to elongated canine chromosome preparations and/or by reference to interphase FISH analysis. Additional information may be found at .

### Alignment of dog BAC clones to orthologous regions of the human genome

Nineteen 384-well plates of BAC clones from the RPCI-81 library [27] were selected at random, and end-sequence data were obtained from each clone using previously described methods [1]. Paired end-sequences for 1910 clones were masked for repetitive elements and searched against the human genome (NCBI build 31, November 2002, ). For 648 of the BACs (34%), each of the paired end-sequences gave a best hit to human genomic locations that are separated by 50–500 Kb, and aligned head-to-head (mean span of human genomic DNA = 191 Kb). The remaining 1262 BAC-end sequences were searched against scaffolds of the 1.5x assembly [2] using wu-blastn (matrix = identity, W = 40) to identify scaffold sequences that contained at least short overlaps (40 bases) of identical sequence. For 954 of the 1262, hits were detected for both of the paired end-sequences. The homologous scaffold sequences were trimmed to remove any sequence that extended beyond 5 Kb from the region of alignment. They were then masked for repetitive elements, and searched against the human genome using wu-blastn (E<0.1). Again, only the best hit was considered. For 604 of the BACs (32% of the original sample), the paired scaffolds gave the best hits to genomic locations that are separated by 50–500 Kb (mean = 202 Kb), and aligned with their component BAC-end sequences in a head-to-head orientation. Altogether a total of 1252 (648+ 604) canine paired BAC-end sequences demonstrated significant hits with the human sequence.

Accession numbers, PCR conditions, primers and BAC-end sequences are available for all markers at:  and .

## Abbreviations

BAC-Bacterial Artificial Chromosome

FISH-Fluorescence *in situ *Hybridization

PCR-Polymerase Chain Reaction

RH-Radiation Hybrid

CGH-Comparative Genomic Hybridization

cR-centiRays

Mb-megabases

Kb-kilobases

Bp-base pair

Min.-minutes

E. Coli-Escherichia coli

SOM-Supporting Online Material

DAPI-Diamidino-2-phenylindole

CS-Conserved Segments

CSO-Conserved and Ordered Segments

TIGR-The Institute for Genomics Research

FHCRC-Fred Hutchinson Cancer Research Center

## Authors' contributions

MB, RT, AS, and RH all contributed to the FISH mapping aspects of the manuscript, including isolation of BAC DNA samples, generation of canine chromosome preparations, probe labeling and purification, fluorescence *in situ *hybridization, and microscopy. MB performed all the cytogenetic analysis to generate detailed probe ordering. MB and RT completed the merging of RH and FISH data, and prepared the drawing of Figure 2 for both the manuscript and web sites. MB also wrote and edited relevant sections of the manuscript. CH did the statistical analysis for the localization of the 4249 markers on the radiation hybrid map and the integration of the FISH and RH maps. In addition, CH constructed Figure 1 (paper and web sites) and Table 1 and implemented the accompanying web site. GGM and EFK organized and oversaw the BAC-end sequencing, including data production and analysis, with CMF overseeing the ultimate effort at TIGR. Markers were RH-mapped in Rennes, France by EC and GE, and in Seattle, Washington, USA by HGP, GB, LS, and TDL. The Seattle data were checked, duplicated, data-entered and generally overseen by TDL. TDL also drafted portions of the methods section of the manuscript. RG computed the marker sequences using BLAST software against the human sequence to identify orthologoussequences and synteny conservation. RG also drew some of the chromosomes for Figures 1 (web sites). Efforts in Rennes, France were overseen by CA and FG, including analysis, quality control, supervision, and portions of manuscript production. Efforts in Seattle, Washington, USA were overseen by EAO including experimental design, quality control, supervision, and portions of manuscript production. Individual and joint grants funding this work were written by and awarded to EAO, MB, and FG. All authors read and approved the final manuscript.

## Links

**FISH Information**: 

**RH Map Information**:  and .
